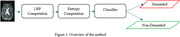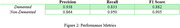# Enhanced Texture Analysis using Quantum Entropy Local Binary Pattern (QELBP) for Early Alzheimer’s Disease Detection on a Large‐Scale MRI Dataset

**DOI:** 10.1002/alz.095106

**Published:** 2025-01-09

**Authors:** Vinu Sherimon, Abraham Varghese

**Affiliations:** ^1^ University of Technology and Applied Sciences, Muscat, Muscat Oman

## Abstract

**Background:**

Recent breakthroughs in Quantum Calculus (QC) have given new opportunities for texture analysis. Motivated by QC, QELBP examines subtle variations in grayscale within MRI, which may provide early signs of textural changes associated with AD.

**Method:**

13500 images were extracted from 150 images (90 slices/patient). Texture descriptors LBP and QELBP were used to extract texture features from the images. Quantum entropy measures the uncertainty in the distribution of LBP. Higher entropy means a more varied texture, while lower entropy points a more uniform pattern. AD is often linked with specific brain changes, such as atrophy, reduced gray matter, and other textural anomalies.

**ALGORITHM**

**Input**:

Grayscale MR Image of size 224×224

Radius R

Classifier

**Output**:

Classified as demented or non‐demented

**Steps**:

1. *Initialize Parameters*: Radius R, and number of points P

2. *Divide Image into non‐overlapping circular blocks of radius R*

3. *Compute LBP for each block in the image*

LBP (x_c_, y_c_) = ∑_i = 0 to P‐1_ s (g_i_ ‐ g_c_) 2^i^, s(x) = 1, x ≥0; 0, x<0, (x_c_, y_c_)‐coordinates of the central pixel, P‐number of surrounding pixels, g_c,_g_i_ are the gray values of central and surrounding pixels.

4. *Calculate Entropy for each block*: ∑ _i = 1 to N_ (P_i_ log_2_P_i_) P_i_ is the probability of i^th^ pattern and concatenate it as a single vector. It represents the textural randomness of the image.

5. *Train a classifier using labeled data to distinguish between MRI categories using entropy as input features*.

**Result:**

Among multiple ML models, Linear Discriminant Analysis (LDA) model achieved a precision of 0.938 for demented and a recall of 0.950 for non‐demented. This high precision and recall suggest that the LDA model aptly identifies demented and seldom mislabels non‐demented, featuring the potential of QELBP in early AD detection.

**Conclusion:**

The superior performance of the LDA model underscores the promise of QELBP in early AD identification. Future studies should examine the ability of this method to distinguish between dementia subtypes. Moreover, fusing these textural features with additional biomarkers could enhance diagnostic accuracy.